# Increased end-stage renal disease risk in age-related macular degeneration: a nationwide cohort study with 10-year follow-up

**DOI:** 10.1038/s41598-022-26964-8

**Published:** 2023-01-05

**Authors:** Wonyoung Jung, Junhee Park, Hye Ryoun Jang, Junseok Jeon, Kyungdo Han, Bongseong Kim, Je Moon Yoon, Dong Hui Lim, Dong Wook Shin

**Affiliations:** 1 Department of Family Medicine/Supportive Care Center, Samsung Medical Center, Sungkyunkwan University School of Medicine, 81 Irwon-Ro, Gangnam-Gu, Seoul, 06351 Republic of Korea; 2 Department of Medicine, Sungkyunkwan University School of Medicine, Seoul, Republic of Korea; 3 Division of Nephrology, Department of Medicine, Samsung Medical Center, Sungkyunkwan University School of Medicine, Seoul, Republic of Korea; 4 Department of Statistics and Actuarial Science, Soongsil University, Seoul, Republic of Korea; 5 Department of Ophthalmology, Samsung Medical Center, Sungkyunkwan University School of Medicine, 81 Irwon-Ro, Gangnam-Gu, Seoul, 06351 Republic of Korea; 6 Department of Clinical Research Design & Evaluation, Samsung Advanced Institute for Health Science & Technology (SAIHST), Sungkyunkwan University, 81 Irwon-Ro, Gangnam-Gu, Seoul, 06351 Republic of Korea

**Keywords:** End-stage renal disease, Macular degeneration

## Abstract

Common etiologies between age-related macular degeneration (AMD) and kidney disease advocate a close link between AMD and end-stage renal disease (ESRD). However, the risk of ESRD in people with AMD was not reported. Here, we investigated the association between AMD and the risk of ESRD by using a nationwide, population-based cohort data in Korea. 4,206,862 participants aged 50 years or older were categorized by presence of AMD and visual disability. Risk of ESRD was the primary outcome. Cox regression hazard model was used to examine the hazard ratios (HRs) with adjustment for potential confounders. Stratified analyses by age, sex, baseline kidney function, and cardiometabolic comorbidities were performed. During the mean 9.95 years of follow-up, there were 21,759 incident ESRD events (0.52%). AMD was associated with 33% increased risk of ESRD (adjusted HR [aHR] 1.33, 95% confidence interval [CI] 1.24–1.44), and the risk was even higher when accompanied by visual disability (aHR 2.05, 95% CI 1.68–2.50) than when not (aHR 1.26, 95% CI 1.17–1.37). Age, baseline kidney function, and cardiometabolic comorbidities significantly interact between AMD and the risk of ESRD. Our findings have clinical implications on disease prevention and risk factor management of ESRD in patients with AMD.

## Introduction

End-stage renal disease (ESRD) is a significant public health problem. The worldwide number of patients who required renal replacement therapy (RRT) was 2·61 million in 2010 and is estimated to increase to 5·43 million in 2030^1^. RRT is expensive, and easy access to healthcare facility is demanded for people who requires RRT. In addition, patients with ESRD show a higher mortality than the general population^2^.

Age-related macular degeneration (AMD) is a progressive and degenerative retinal disease that can influence central vision^3^. It is the leading cause of visual disability in developed countries, and the number of individuals with AMD worldwide is expected to reach 288 million by 2040^4^. Therefore, addressing the comorbid conditions and health problems in AMD population is highlighted.

From earlier epidemiologic findings of AMD studies (the Blue Mountains Eye study^5^, Beaver Dam Eye Study^6^, and the US NHANES III^7^), and more recent investigation of genetic polymorphisms in complement factor H (CFH)^8–11^, emerging evidence has suggested an association between renal impairment and AMD (Supplementary Table S1). The common etiologies between chronic kidney disease (CKD) and AMD, including cardiometabolic risk factors (age, smoking, hypertension, and dyslipidemia^12–19^), pathogenic mechanisms (atherosclerosis, inflammation, and oxidative stress^20^) and similar structural, and genetic pathways between kidney and eye^21,22^, advocate a close link between AMD and CKD.

While most epidemiologic studies have established the association between CKD and the risk of AMD, to our best knowledge, the association between AMD and the risk of ESRD was not reported. In addition, the presence of visual disability was not considered in previous studies on the association of AMD and ESRD. Globally, the number of people with blind or with moderate to severe visual impairment caused by macular disease is approximately 2.1 million^23^. Visual disability is associated with broad range of chronic conditions, including cardiometabolic, neuropsychiatric, and musculoskeletal disorders^24–26^. Therefore, we conducted a retrospective, nationwide, population-based cohort study to investigate the risk of ESRD in patients with AMD, considering the visual disability status.

## Results

### Baseline characteristics

Of the total 4,206,862 participants enrolled in the final analysis, 53,617 participants (1.27%) had AMD at baseline, and 3858 participants (7.20% of the AMD group) had visual disability. The descriptive characteristics of the study population, classified by the presence of AMD and further by visual disability, are summarized in Table 1.

**Table 1:** Baseline characteristics of the study population.

	Age-related macular degeneration		*P*-value	Age-related macular degeneration		*P*-value
	Absent (N = 4,153,245)	Present (N = 53,617)		Without VD (N = 49,759)	With VD (N = 3,858)	
Mean age, years	60.6 ± 8.3	67.4 ± 8.4	< 0.001	67.4 ± 8.5	67.9 ± 8.3	< 0.001
Sex, male, No. (%)	2,013,798 (48.5)	22,783 (42.5)	< 0.001	20,927 (42.1)	1,856 (48.1)	< 0.001
Smoking, No. (%)			< 0.001			< 0.001
Non-smoker	2,764,235 (66.6)	39,328 (73.4)		36,589 (73.5)	2,739 (71.0)	
Ex-smoker	659,802 (15.9)	8,514 (15.9)		7,890 (15.9)	624 (16.2)	
Current smoker	729,208 (17.6)	5,775 (10.8)		5,280 (10.6)	495 (12.8)	
Alcohol consumption, No. (%)			< 0.001			< 0.001
Non	2,699,203 (65.0)	40,658 (75.8)		37,709 (75.8)	2,949 (76.4)	
Mild	1,188,326 (28.6)	10,903 (20.3)		10,148 (20.4)	755 (19.6)	
Heavy	265,716 (6.4)	2,056 (3.8)		1,902 (3.8)	154 (4.0)	
Regular physical activity, No (%)	880,807 (21.2)	11,137 (20.8)	0.014	7,295 (20.3)	566 (20.8)	0.047
Anthropometrics						
Body mass index, kg/m^2^	24.1 ± 3.0	24.0 ± 3.1	< 0.001	24.0 ± 3.0	23.9 ± 3.1	< 0.001
WC, cm	82.2 ± 8.3	82.9 ± 8.4	< 0.001	82.8 ± 8.3	83.1 ± 8.4	< 0.001
Systolic BP, mmHg	126.5 ± 15.8	128.2 ± 15.9	< 0.001	128.3 ± 15.9	128.0 ± 16.3	< 0.001
Diastolic BP, mmHg	77.9 ± 10.2	77.5 ± 10.0	< 0.001	77.5 ± 10.0	77.5 ± 10.2	< 0.001
Comorbidity						
Hypertension, No. (%)	1,916,948 (46.2)	32,345 (60.3)	< 0.001	30,065 (60.4)	2,280 (59.1)	< 0.001
Diabetes Mellitus, No. (%)	633,576 (15.3)	12,464 (23.3)	< 0.001	11,509 (23.1)	955 (24.8)	< 0.001
Dyslipidemia, No. (%)	1,174,483 (28.3)	18,834 (35.1)	< 0.001	17,509 (35.2)	1,325 (34.3)	< 0.001
Laboratory findings						
Glucose, fasting, mg/dL	102.1 ± 27.4	104.3 ± 29.7	< 0.001	104.3 ± 29.6	105.2 ± 31.3	< 0.001
eGFR, mL/min/1.73 m^2^	82.8 ± 33.3	79.7 ± 34.9	< 0.001	79.8 ± 34.9	79.3 ± 34.9	< 0.001
Total cholesterol, mg/dL	201.0 ± 38.3	198.9 ± 39.3	< 0.001	198.9 ± 39.3	198.0 ± 39.6	< 0.001
Triglycerides, mg/dL	121.3 (121.2–121.4)	121.1 (120.5–121.6)	0.790	120.8 (120.3–121.4)	123.7 (121.7–**–**125.7)	0.022
HDL-C, mg/dL	55.6 ± 31.2	55.2 ± 34.0	0.006	55.3 ± 33.9	54.3 ± 35.0	0.010
LDL-C, mg/dL	119.0 ± 39.5	118.0 ± 39.7	< 0.001	118.0 ± 39.7	117.5 ± 40.5	< 0.001
Hemoglobin, mg/dL	13.7 ± 1.5	13.4 ± 1.5	< 0.001	13.4 ± 1.5	13.3 ± 1.5	< 0.001
Urban residency, No. (%)	1,882,203 (45.3)	22,059 (41.1)	< 0.001	20,469 (41.1)	1,590 (41.2)	< 0.001
Income of lowest 20%, No. (%)	891,189 (21.5)	9,602 (17.9)	< 0.001	8,862 (17.8)	740 (19.2)	< 0.001
Charlson comorbidity index	1.2 ± 1.3	1.9 ± 1.6	< 0.001	1.9 ± 1.6	1.9 ± 1.6	< 0.001
Visual disability, No. (%)	40,287 (1.0)	3,858 (7.2)	< 0.001			

Data are expressed as mean ± standard deviation or number (%).

*VD* visual disability; *WC* waist circumference; *BP* blood pressure; *eGFR* estimated glomerular filtration rate; *HDL-C* high-density lipoprotein cholesterol; *LDL-C* low-density lipoprotein cholesterol.

At baseline, the AMD group was older with a high proportion of women and non-smokers than non-AMD group, or participants without AMD; the average age at baseline was 67.4 ± 8.4 years, 42.5% were men and 10.8% were current smokers. The AMD group had a higher prevalence of hypertension, diabetes mellitus, and dyslipidemia, but lower levels of eGFR and hemoglobin than the non-AMD group (all *P* < 0.001). At baseline, the AMD with visual disability group was older with a higher proportion of men and current smokers than AMD without visual disability group; the average age at baseline was 67.9 ± 8.3 years, 48.1% were men, and 12.8% were current smoker. The AMD with visual disability group had a lower prevalence of hypertension, and dyslipidemia than the AMD without visual disability group (all *P* < 0.001).

### Incidence of ESRD by the presence of AMD and visual disability

**Figure 1:**
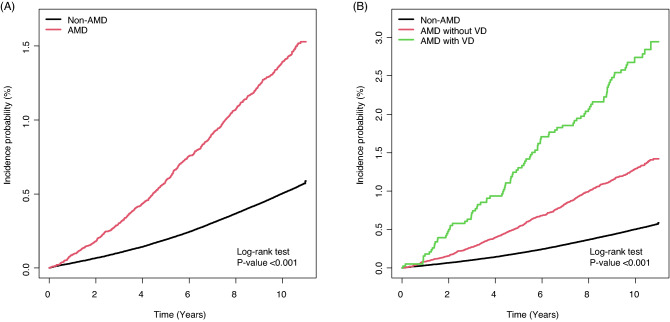
Kaplan–Meier curves displaying the estimated incidence probability of ESRD according to the presence of AMD, and VD. Kaplan–Meier analysis estimated an increased risk of ESRD in people with AMD (**A**); In addition, compared to non-AMD group, an increased risk of ESRD among the AMD without VD group, and an even higher risk of ESRD among the AMD and VD group were observed (**B**). AMD, age-related macular degeneration; VD, visual disability.

During the mean 9.95 years of follow-up, there were 21,759 (0.52%) incident ESRD events. AMD was associated with a 33% increased risk of ESRD (aHR 1.33, 95% CI 1.24–1.44, *P* < 0.001). Further analysis that specified AMD by visual disability status demonstrated a 1.26-fold higher risk of ESRD in AMD without visual disability (aHR 1.26, 95% CI 1.17–1.37) and 2.05-fold higher risk of ESRD in AMD with visual disability (aHR 2.05, 95% CI 1.68–2.50). In addition, there was a significant trend in the risk of ESRD among non-AMD, AMD without visual disability, and AMD with visual disability groups (*P* for trend < 0.001) (Table 2 and Fig. 1).

**Table 2:** Association between AMD and the risk of ESRD according to visual disability.

	Subjects (N)	Case (N)	IR per 1,000 person-years	Model 1 (Crude) HR (95% CI)	Model 2 aHR (95% CI)	Model 3 aHR (95% CI)	Model 4 aHR (95% CI)
Non-AMD	4,153,245	21,042	0.50	1 (Ref.)	1 (Ref.)	1 (Ref.)	1 (Ref.)
AMD	53,617	717	1.40	**2.77 (2.57–2.98)**	**2.00 (1.85–2.15)**	**1.98 (1.84–2.13)**	**1.33 (1.24–1.44)**
*P*-value				< 0.001	< 0.001	< 0.001	< 0.001
AMD without VD	49,759	619	1.30	**2.57 (2.37–2.78)**	**1.86 (1.72–2.02)**	**1.85 (1.70–2.00)**	**1.26 (1.17–1.37)**
AMD with VD	3,858	98	2.73	**5.42 (4.45–6.61)**	**3.69 (3.03–4.51)**	**3.60 (2.95–4.40)**	**2.05 (1.68–2.50)**
^†^*P* for trend				< 0.001	< 0.001	< 0.001	< 0.001

*IR* incidence rate; *PY* person-years; *HR* hazard ratio; *aHR* adjusted hazard ratio; *CI* confidence interval.

^†^P for trend was calculated by the (adjusted) hazard ratios among non-AMD, AMD without VD and AMD with VD groups.

Model 2: adjusted for age and sex.

Model 3: Model 2 + income, area of residence, body mass index, smoking, alcohol consumption, and regular physical activity.

Model 4: Model 3 + fasting serum glucose level, serum hemoglobin level, estimated glomerular filtration rate, hypertension, diabetes mellitus, dyslipidemia, and Charlson comorbidity index.

Significant values are in bold.

### Stratified analysis

In stratified analyses according to age, sex, and comorbidity (any of hypertension, diabetes mellitus, or dyslipidemia), the AMD with visual disability group had a markedly higher risk of developing ESRD than controls (Table 3). In addition, the impact of AMD with visual disability on developing ESRD was more prominent among individuals aged 50–64 than those aged 75 or older (aHR 3.17 vs. 1.62; *P* for interaction < 0.001) among individuals with eGFR of same or above 60 mL/min/1.73 m^2^ than eGFR of less than 30 mL/min/1.73 m^2^ (aHR 2.62 vs. 1.63; *P* for interaction < 0.001) and among individuals without comorbidity than those with comorbidity (aHR 5.08 vs. 1.96; *P* for interaction < 0.001).

**Table 3:** Risk of ESRD by AMD and visual disability according to age, sex, baseline kidney function, and cardiometabolic comorbidities.

			Subjects (N)	End-stage renal disease			
				Case (N)	IR per 1,000 person-years	aHR^†^ (95% CI)	P for interaction
Age group	50**–**64	Non-AMD	2,886,066	10,564	0.36	1 (Ref.)	< 0.001
		AMD without VD	17,789	234	1.30	1.97 (1.73–2.24)	
		AMD with VD	1,278	30	2.34	3.17 (2.21–4.54)	
	65**–**74	Non-AMD	1,003,820	8,220	0.84	1 (Ref.)	
		AMD without VD	22,305	285	1.32	1.10 (0.98–1.24)	
		AMD with VD	1,767	48	2.90	1.85 (1.40–2.46)	
	75-	Non-AMD	263,359	2,258	1.03	1 (Ref.)	
		AMD without VD	9,665	100	1.24	0.89 (0.73–1.09)	
		AMD with VD	813	20	3.05	1.62 (1.04–2.51)	
Sex	Male	Non-AMD	2,013,798	13,060	0.66	1 (Ref.)	0.076
		AMD without VD	20,927	346	1.79	1.25 (1.13–1.40)	
		AMD with VD	1,856	57	3.46	1.73 (1.33–2.25)	
	Female	Non-AMD	2,139,447	7,982	0.37	1 (Ref.)	
		AMD without VD	28,832	273	0.96	1.28 (1.13–1.44)	
		AMD with VD	2,002	41	2.11	2.76 (2.03–3.75)	
Baseline kidney function (eGFR*^)^	≥ 60	Non-AMD	3,729,357	8,874	0.24	1 (Ref.)	< 0.001
		AMD without VD	41,538	240	0.60	1.66 (1.46–1.89)	
		AMD with VD	3,173	30	1.00	2.62 (1.83–3.74)	
	30 ≥ , < 60	Non-AMD	346,472	8,301	2.53	1 (Ref.)	
		AMD without VD	7,230	256	3.92	0.93 (0.82–1.06)	
		AMD with VD	616	55	10.40	1.85 (1.42–2.41)	
	< 30	Non-AMD	77,416	3867	5.16	1 (Ref.)	
		AMD without VD	991	123	14.28	1.62 (1.35–1.94)	
		AMD with VD	69	13	25.99	1.63 (0.95–2.80)	
Any of hypertension, diabetes mellitus, or dyslipidemia	No	Non-AMD	1,628,379	1,721	0.10	1 (Ref.)	< 0.001
		AMD without VD	12,995	39	0.30	2.42 (1.76–3.32)	
		AMD with VD	1,052	7	0.69	5.08 (2.42–10.68)	
	Yes	Non-AMD	2,524,866	19,321	0.78	1 (Ref.)	
		AMD without VD	36,764	580	1.66	1.22 (1.13–1.33)	
		AMD with VD	2,806	91	3.52	1.96 (1.59–2.41)	

*eGFR* estimated glomerular filtration rate; *VD* visual disability.

*Units: mL/min/1.73 m^2^.

^†^ adjusted for age and sex, income, area of residence, body mass index, smoking, alcohol consumption, and regular physical activity, fasting serum glucose level, serum hemoglobin level, estimated glomerular filtration rate, hypertension, diabetes mellitus, dyslipidemia, and Charlson comorbidity index.

## Discussion

To our best knowledge, this is the first large-scale nationwide study to establish that AMD was associated with a higher risk of ESRD, after considering various factors including age, sex, BMI, lifestyle behaviors (smoking, alcohol consumption, physical activity), and comorbidities (hypertension, diabetes mellitus, dyslipidemia). Notably, the risk was even higher for AMD patients with visual disability, reaching a nearly two-fold risk. This effect was noticeable in individuals 50–64 years old, individuals with eGFR ≥ 60 mL/min/1.73 m^2^, and those without cardiometabolic comorbidities.

Findings from numerous previous epidemiologic studies have suggested the association between renal impairment and AMD^5–7,27–31^. As no previous studies have examined the risk of ESRD in people with AMD, these findings provided initial background for our research with shared risk factors and pathophysiology between two diseases.

Our findings are supported by several plausible biologic mechanisms. First, the kidney and eye share common developmental pathways and have similar anatomical structures. Both the PAX and WT1 pathways are important in the embryogenesis of the kidney and retina^22^. Furthermore, both choroid and glomerulus have extensive vascular networks that are similar in structure, and the retinochoroidal junction resembles the glomerular filtration barrier^32^. In addition, the renin–angiotensin–aldosterone system is present in both eye and kidney^21^.

Second, AMD and ESRD share cardiovascular risk factors. Risk factors for AMD including old age, current smoking, and hypertension^33^, and pathogenic mechanisms of AMD development including atherosclerosis and oxidative stress^34,35^ are all implicated in the development of ESRD, which helps support our findings.

Third, growing evidence from genetic studies suggests that dysregulation of the complement system has a pathogenic role in both AMD^9,36,37^ and kidney diseases including atypical hemolytic uremic syndrome (aHUS)^38,39^, membranoproliferative glomerulonephritis (MPGN)^40^, IgA nephropathy^41^, and DM nephropathy^42^. Complement factor H (CFH) is a soluble complement regulator that is essential for controlling the alternative complement pathway and protects against oxidative stress. A genome-wide association study revealed a higher risk of AMD in individuals with a genetic polymorphism (Y402H) in the CFH gene^9,11^ of which locus is in a region that binds C-reactive protein. Therefore, we speculate that from the genetic polymorphism of CFH Y402H, reduced binding of C-reactive protein to the CFH protein limits the function of CFH, which results in the development of AMD and kidney disease.

Notably, we clearly demonstrated that the risk of ESRD was higher in AMD patients with visual disability (*P* for trend < 0·001). In our study, we examined the influence of visual disability status among AMD patients, which represents a novelty of our study. visual disability in AMD patients occurs when early AMD progresses to late AMD, which is classified into neovascular AMD and geographic atrophy. Therefore, advances in the pathogenic mechanism of AMD, especially regarding atherosclerosis, oxidative stress, and cardiovascular risks factors, may reinforce the risk of ESRD incidence.

Visual impairment is associated with physical inactivity^43–45^ and may have additional adverse results for people with visual disability. Previous nationwide studies have demonstrated that lower physical activity levels are associated with lower kidney function^46^ and the incidence of ESRD^47^. Visual impairment can also have adverse functional consequences including social isolation, restriction of daily activities, poor quality of life, and frailty^26,48–55^, especially in the elderly population; this further significantly affects nutritional status, with higher prevalence of obesity and malnutrition in patients with visual impairment^56^. Moreover, individuals with visual disability may experience great difficulty in accessing healthcare services. Therefore, appropriate management of risk factors would be challenging. For individuals with severe central vision loss, which is typical in advanced AMD, their reduced ability to read may lead to poor compliance or even overdose problems that possibly affect renal function. From these multiple components that interplay between visual impairment and adverse health outcomes, our findings that AMD patients with visual disability have much higher risk of ESRD than non-AMD group or even AMD patients without visual disability may be explained. A further prospective study is required to elucidate the chronological order in AMD and visual disability.

Intriguingly, our findings showed that the risk of ESRD was higher in AMD patients with younger age (50–64 years old), preserved renal function (eGFR ≥ 60 mL/min/1.73 m^2^), and without underlying cardiometabolic comorbidity, all of which are considered to be low risk factors for ESRD. This suggests that AMD is more strongly associated with ESRD risk when there are no other risk factors of AMD, which is a common finding in this kind of stratified analyses.

Several limitations should be considered in interpreting our findings. First, we defined AMD patients based on the diagnostic code (ICD-10) from the national database. In the clinical setting, the diagnosis of AMD is usually made on the basis of ophthalmoscopy during ophthalmologic examination, and a false positive diagnosis is not common. However, AMD is often underdiagnosed if symptoms are not severe, or some people may regard it as a normal aging process that patients do not seek health care services. Therefore, some AMD patients might have been categorized into the non-AMD group, making the observed association weaker than the true association. Second, considering the ethnic difference in genetic factors related to AMD^57–60^, our results cannot be generalized to other ethnic groups. Lastly, other possible confounders including nutrition and dietary factors, which might be associated with both AMD and ESRD^61,62^ were not controlled. Therefore, careful consideration is required to extrapolate the results to other groups.

We demonstrated that AMD is associated with an increased risk of ESRD. Remarkably, much higher risks of ESRD were found for AMD with visual disability. Our findings have clinical implications on disease prevention and risk factor management of ESRD in patients with AMD, and visual disability, suggesting shared risk factors and pathogenic mechanisms between AMD and ESRD.

## Materials and methods

### Data source and study setting

The national health insurance service (NHIS) provides mandatory universal coverage to 97% of the Korean population; the remaining 3% are Medicaid beneficiaries. For every individual 40 years old or above, the NHIS provides a biennial national health screening program that consists of a self-questionnaire on health behavior (smoking, drinking, and past medical history), anthropometric measurements (blood pressure, body mass index), and laboratory test findings (fasting glucose, serum lipid levels). The NHIS database consists of an eligibility database (age, sex, disability type and severity, socioeconomic variables, income level, type of eligibility), a medical treatment database (based on medical bills claimed by medical service providers for their medical expense claims), and therefore this nationwide database is widely used in epidemiologic studies in Korea^63^.

### Study population

Among 4,470,729 participants (age ≥ 50) who underwent general health screenings in 2009, we first excluded 11,515 participants who had ESRD at the baseline. Next, 252,352 participants with missing data on at least one variable were excluded. Finally, a total of 4,206,862 participants were included in the analyses.

### Definition of age-related macular degeneration

Individuals with AMD were identified on the basis of the International Classification of Diseases, 10th Revision (ICD-10) code for AMD (H353) by an ophthalmologist within one year before the health screening examination. This code includes early, intermediate, atrophic (geographic atrophy), and neovascular AMD. This operational definition of AMD was used in previous epidemiologic studies on AMD^64,65^.

### Definition of visual disability

According to the National Disability Registration System in Korea, visual disability refers to a visual loss or visual field defect. Registration for disability requires the submission of the validated documentation of the results of disability diagnosis by a specialist physician^66^. A best-corrected visual acuity (BCVA) of 20/100 or less using the Snellen visual acuity chart is a minimal requirement to register for visual disability. In Korea, the severity of disability is graded from 1 (most severe) to 6 (least severe) (Supplementary Table S2). Several welfare benefits, including Disability Pension, have been determined using the severity^67^. Therefore, since almost every individual with disabilities applies for the registration, we were able to identify almost all individuals with visual disability in the National Disability Registration System in Korea.

### Study outcomes and follow-up

The endpoints of the study were incident ESRD. Newly diagnosed ESRD was defined by the combination of an ICD-10 code (N18-19, Z49, Z94.0, and Z99.2) and a special code (V code) that indicates required hemodialysis (V001), peritoneal dialysis (V003), or kidney transplantation (V005). All medical expenses for dialysis are reimbursed using the Korean Health Insurance Review and Assessment Service database. These patients are also registered as special medical aid beneficiaries. Therefore, we were able to identify every patient with ESRD in the entire Korean population and to analyze the data for all patients with ESRD who underwent dialysis^68^. Codes for treatment or medical expense claims included V005 for kidney transplantation, V001 for hemodialysis, and V003 for peritoneal dialysis. We excluded individuals without previous CKD who had a transplant or dialysis code on the same date as an acute renal failure code. Subjects on continuous renal replacement therapy or acute peritoneal dialysis were also excluded. The participants were followed from the date of health screening examination in 2009 to the date of incident ESRD, death, or until the end of the study period (December 31, 2019), whichever came first.

### Covariates

During the health screening, participants provided information on lifestyle behaviors using standardized questionnaires^69^. Smoking status was categorized as non-, ex-, and current smoking. Alcohol consumption was categorized as none, mild, and heavy drinking; heavy drinking was defined as ≥ 30 g of alcohol consumption per day. Regular physical activity was defined as strenuous exercise ≥ one time/week for at least 20 min per session. Household income was dichotomized by the lowest 20 percentile according to the health insurance premium (in the social health insurance system in Korea, insurance premium is determined by income status, not by health status). Body mass index (BMI) was calculated as weight in kilograms divided by height in meters squared.

Fasting serum glucose level, serum hemoglobin level, and estimated glomerular filtration rate (eGFR) were also assessed. Hypertension was defined as any of the following: systolic blood pressure ≥ 140 mmHg; diastolic blood pressure ≥ 90 mmHg; or treatment with an antihypertensive medication that was linked to hypertension ICD-10 codes (I10-I13 and I15) and resulted in at least one claim in a year. Diabetes mellitus was defined as a blood glucose level ≥ 126 mg/dL or history of a hypoglycemic medication prescription that was linked to diabetes mellitus ICD-10 code (E11-E14) and resulted in at least one claim in a year. Dyslipidemia was defined as total cholesterol ≥ 240 mg/dL or history of a lipid-lowering medication that was associated with an ICD-10 code (E78). Charlson comorbidity index (CCI) was calculated based on diagnosis code^70^.

### Statistical analysis

The comparison of baseline characteristics by the presence of AMD and visual disability was conducted using t-test for continuous variables or the chi-square test for categorical variables. The incidence rates of ESRD were calculated by the number of incident cases divided by 100,000 person-years. Cox hazard regression model was used to examine the hazard ratios (HRs) of ESRD. Multivariable analyses were adjusted for age and sex in model 2 and for age, sex, household income, area of residence, body mass index, smoking, alcohol consumption, and regular exercise in model 3. In our final model (model 4), fasting serum glucose level, serum hemoglobin level, estimated glomerular filtration rate (eGFR), hypertension, diabetes mellitus, dyslipidemia, and Charlson comorbidity index (CCI) were included in addition to every adjusting variable used in model 3. P for trend was calculated among the hazard ratios of control, AMD without visual disability, and AMD with visual disability. Kaplan–Meier curves were presented as cumulative incidence probabilities of ESRD. Finally, to evaluate the potential effect modification by age, sex, eGFR, and comorbidity status, P for interaction was calculated using stratified analyses.

All statistical analyses were performed using SAS version 9·4 (SAS Institute Inc., Cary, NC, USA). *P* < 0·05 were considered to be statistically significant.

### Ethic statement

This study was reviewed and approved by the Institutional Review Board of Samsung Medical Center (SMC IRB no. 2022-03-060) and waived the requirement to obtain any informed consent because anonymized and de-identified information in compliance with the confidentiality guidelines was used for analyses. This study adhered to the tenets of the Declaration of Helsinki.

## Supplementary Information

Supplementary Information.

## Data Availability

The datasets generated and/or analyzed during the current study are available in the Korean National Health Insurance Sharing Service database repository, (https://nhiss.nhis.or.kr). The datasets used and/or analyzed during the current study available from the NHIS on reasonable request.
