# Supplementary material for: Increased end-stage renal disease risk in age-related macular degeneration: a nationwide cohort study with 10-year follow-up
Source: Sci Rep. 2023 Jan 5;13:183. doi: 10.1038/s41598-022-26964-8 (PMC9814881; doi:10.1038/s41598-022-26964-8)
Supplement: Supplementary file 1 — Supplementary Information. [file 41598_2022_26964_MOESM1_ESM.pdf]

Supplementary Table S1. Summary of previous research on the association between renal impairment and age-related macular degeneration

Author (et al.)	Year	Country	Number of participants	Outcome	Definition of CKD	Main finding
**Chen**	2017	Taiwan	775,077	All, advanced AMD	Mild to moderate CKD (ICD-9 codes: 250.4, 274.1, 283.11, 403.1, 404.2, 404.3, 440.1, 442.1, 447.3, 572.3, 580–588, 593, 642.1, 646.2, and 753.1)	Mild to moderate CKD was associated with all AMD (OR 1.320; 95% CI 1.218–1.431), and advanced AMD (OR 1.447; 95% CI 1.169–1.791).
**Choi**	2011	Republic of Korea	3,008	Early AMD	eGFR < 60 mL/min/1.73 m^2	CKD was associated with early AMD (OR 1.68; 95% CI 1.04–2.72); peripheral retinal drusen (OR 2.01; 95% CI 1.02–3.99).
**Weiner**	2011	US	Case 865, control 865	Any, early, late AMD	eGFR < 60 mL/min/1.73 m^2 and microalbuminuria	Lower eGFR was associated with late AMD (OR 3.05; 95% CI 1.51–6.13), while albuminuria was not significant.
**Klein**	2009	US (Beaver Dam Eye Study)	4,926	1) Early, late AMD<br>2) AMD progression	1) Serum cystatin C level<br>2) Mild CKD (eGFR 45-60mL/min/1.73 m^2)<br>3) Moderate to severe CKD (eGFR < 45 mL/min/1.73 m^2)	1) Serum cystatin C was associated with early AMD (OR per SD 1.16; 95% CI 1.01–1.35), and exudative AMD (OR per SD 1.42; 95% CI 1.03–1.96), but not with geographic atrophy.<br>2) Mild CKD was associated with 15-year cumulative incidence of early AMD (OR per SD 1.36; 95% CI 1.00–1.86).
**Liew**	2008	Australia (Blue Mountains Eye Study)	1,183	Early AMD	eGFR < 60 mL/min/1.73 m^2	Moderate CKD was associated with early AMD (OR 3.2; 95% CI 1.8–5.7).

Supplementary Table S2. Visual disability criteria in the Republic of Korea

Grade of Visual Disability	Criteria
1 (most severe)	BCVA ≤ 20/1000 in the better eye
2	BCVA ≤ 20/500 in the better eye
3	1) BCVA ≤ 20/320 in the better eye, or 2) bilateral visual field < 5 degrees in all perspectives
4	1) BCVA ≤ 20/200 in the better eye, or 2) bilateral visual field < 10 degrees in all perspectives
5	1) BCVA ≤ 20/100 in the better eye, or 2) Decrease in bilateral visual field of 50% or more from the normal
6 (least severe)	BCVA ≤ 20/100 in the worse eye
